# Disease modulation by TIV vaccination during secondary pneumococcal infections in influenza-infected mice

**DOI:** 10.1128/jvi.01774-25

**Published:** 2025-12-29

**Authors:** Juan García-Bernalt Diego, Javier Arranz-Herrero, Gabriel Laghlali, Eleanor Burgess, Seok-Chan Park, Gagandeep Singh, Lauren A. Chang, Prajakta Warang, Moataz Noureddine, Jordi Ochando, Estanislao Nistal-Villan, Michael Schotsaert

**Affiliations:** 1 Department of Microbiology, Icahn School of Medicine at Mount Sinai 5925, New York, New York, USA; 2 Global Health and Emerging Pathogens Institute, Icahn School of Medicine at Mount Sinai 5925, New York, New York, USA; 3 Microbiology Section, Dpto. CC, Farmacéuticas y de la Salud, Facultad de Farmacia, Universidad San Pablo-CEU, CEU Universities, Madrid, Spain; 4 Institute of Applied Molecular Medicine-Nemesio Díez (IMMA-ND), Department of Basic Medical Sciences, Facultad de Medicina, Universidad San Pablo-CEU, CEU Universities, Urbanización Montepríncipe, Madrid, Spain; 5 National Microbiology Center, National Institutes of Health Carlos III, Madrid, Spain; 6 Department of Pharmaceutics, Ghent University 26656, Ghent, Belgium; 7 Graduate School of Biomedical Sciences, Icahn School of Medicine at Mount Sinai 5925, New York, New York, USA; 8 Department of Oncological Sciences, Icahn School of Medicine at Mount Sinai 5925, New York, New York, USA; 9 Icahn Genomics Institute, Icahn School of Medicine at Mount Sinai 5925, New York, New York, USA; 10 Marc and Jennifer Lipschultz Precision Immunology Institute, Icahn School of Medicine at Mount Sinai 5925, , New York, New York, USA; Emory University School of Medicine, Atlanta, Georgia, USA

**Keywords:** *Streptococcus pneumoniae*, influenza virus, TIV, bacterial coinfection, bacterial superinfection

## Abstract

Secondary bacterial infections can significantly worsen the clinical course of influenza virus infections and are a leading cause of morbidity and mortality during seasonal influenza epidemics. Despite being a vaccine-preventable disease, influenza-related complications from secondary bacterial infections are an important cause of death, particularly among the elderly population. *Streptococcus pneumoniae* (Spn) is the most common agent responsible for influenza-related secondary bacterial infections. Influenza virus vaccination serves as an effective prophylactic strategy for preventing influenza and reducing the burden of influenza-associated pathology, including secondary bacterial infection. However, whether the protective effects of influenza virus vaccination differ in the context of a secondary Spn infection at the level of the host response remains poorly characterized. Here, we present a preclinical mouse model to examine the impact of influenza vaccination in scenarios involving single infections with influenza A virus H1N1 (NC99) or Spn serotype 1; simultaneous infection with both NC99 and Spn (coinfection), or NC99 infection followed by Spn infection seven days later (superinfection). A single dose of trivalent inactivated Influenza vaccine (TIV) is able to decrease infection lethality in both secondary bacterial infection scenarios. Protection is associated with reduction in both viral and bacterial titers, decreased production of pro-inflammatory cytokines, protection of alveolar macrophages, prevention of exacerbated lung neutrophil recruitment, modulation of neutrophil activation status, and induction of lung eosinophil recruitment and activation. These findings underscore the importance of influenza vaccination in modulating disease progression and preventing morbidity and mortality associated with secondary bacterial infections.

**IMPORTANCE:**

In this study, we show that a licensed influenza vaccine not only prevents severe disease upon influenza virus infection but also helps protect against enhanced morbidity due to co- or superinfection with S*treptococcus pneumoniae* in a mouse model. This protection correlates with better control of viral and bacterial titers, as well as with altered host immune responses during bacterial co- and superinfection, characterized by the recruitment of activated granulocytes.

## INTRODUCTION

Complications arising from bacterial secondary infections significantly worsen the clinical course of influenza infections, contributing to high morbidity and mortality rates, even in countries with highly developed healthcare systems (1–4). Despite improvements in our understanding of viral and bacterial pathology, along with better healthcare and extended annual influenza vaccination campaigns, influenza-related complications, especially among elderly patients, continue to claim thousands of lives annually (5). Moreover, estimates suggest that in the context of the last century’s influenza pandemics, more than 40% of deaths were caused by bacterial secondary infections in 1968–69 and 1957–58, and could be as high as 95% in the 1918 pandemic (6). Pathology of bacterial secondary infections has been associated with complications largely attributed to acute inflammation of the lower respiratory tissue. While causal agents of bacterial secondary infections in influenza patients vary, *Streptococcus pneumoniae* (Spn) infections are the most prevalent, accounting for over 30% of cases (7).

Influenza virus infections are characterized by their remarkable capacity to elicit a robust immune responses, leading to acute lung inflammation (8–10). During primary influenza infections, viral replication mainly targets the respiratory epithelium. This results in the recruitment and infiltration of immune cells, which causes direct damage to the host tissue (10, 11). This damage, coupled with the modulation of host immune responses, creates a conducive environment for secondary bacterial infections (11, 12). Understanding the dynamics of concomitant influenza and pneumococcus infections is critical, as they lead to increased disease severity, higher mortality rates, and additional complications in patient management (13, 14). Numerous studies have extensively explored potential mechanisms through which influenza favors bacterial opportunism (12, 15–21). These investigations have illuminated the intricate interplay between influenza and bacterial pathogens, shedding light on phenomena such as epithelial damage (22), dysregulation of alveolar macrophages and neutrophils (13, 23–26), coupled with alterations in their phagocytic capabilities (27, 28) and recruitment (29), or the role of cytokines and chemokines such as IL-10 (26) or CXCL2 (30) driving susceptibility and disease of bacterial secondary infections. Still, research characterizing differential outcomes of bacterial secondary infections that occur at different stages of the influenza infection is lacking. Spn infection kinetics in mice upon lethal intranasal challenge leads to high mortality within 2 to 4 days (31) while influenza infection takes 5 to 7 to reach peak morbidity and mortality (32), which is coupled with a significant depletion of alveolar macrophages in lung tissue and bronchoalveolar fluid (33). Thus, it is to be expected that a secondary bacterial infection occurring within the first 48 h of the influenza infection (coinfection) presents a different outcome to one occurring 5 to 7 days later (superinfection).

Currently, vaccination is the cornerstone of prevention plans against influenza epidemics. Licensed influenza vaccines contain either inactivated, live attenuated influenza viruses or recombinantly produced hemagglutinin (HA). Most inactivated vaccines consist of split viruses or subunit influenza antigens. The standard trivalent inactivated influenza vaccine (TIV) is a non-adjuvanted subunit vaccine composed of hemagglutinin molecules from H1N1 and H3N2 influenza A subtypes and one influenza B (Victoria or Yamagata clade) (34, 35). Given the variable efficacy of influenza vaccination depending on the year and antigenic match between vaccines and circulating viruses, it is interesting to investigate the extent to which influenza vaccination approaches and efficacy contribute to the prevention of complications related to bacterial secondary infections, which has been partially addressed in surveillance studies (36, 37), but is still poorly characterized mechanistically. We have previously shown that the administration of TIV protects from mortality after *Staphylococcus aureus* (Sa) bacterial superinfection in a mouse preclinical model, resulting in reduced lung bacterial titers, limited alveolar macrophage loss, and reduced infiltration of inflammatory monocytes in the lung (38). Other research groups have highlighted that the protection against disease during Sa superinfection by influenza vaccines highly depends on the immune skewing of the host. When the vaccine is administered with Th1-inducing adjuvant formulations (MF59 plus CpG), superior protection is achieved than when Th1/2 (MF59) or Th17 inducers (LTK63) are used (39). Focusing on *Streptococcus*, subcutaneous immunization with formalin-inactivated influenza A vaccine or intranasal immunization with influenza A vaccine and cholera toxin protected more than 75% of mice from death by lethal influenza A-*Streptococcus pyogenes* superinfection (40).

To address these research gaps, we present in this manuscript a preclinical C57BL/6 mice model evaluating the multifaceted interactions between influenza virus and Spn, with two different infection regimes: (i) coinfection, defined as a simultaneous infection with a sublethal dose of A/NewCaledonia/20/1999 Influenza virus (NC99, H1N1) and Spn ATCC6301 serotype 1 strain, a highly common and invasive strain in children and in young adults (41) or (ii) superinfection, defined as an initial infection with NC99 followed, 1 week later, with a Spn infection. Both coinfection and superinfection were characterized by lung viral and bacterial kinetics, as well as immune profiling of lung myeloid populations and cytokine/chemokine production. Additionally, we investigated the potential role of a suboptimal TIV vaccination dose in the protection from morbidity and mortality in the context of bacterial secondary infections, characterizing immune responses from both humoral and cellular perspectives.

## RESULTS

### A single TIV dose significantly decreased murine morbidity and mortality in Spn coinfection and superinfection

**Fig 1:**
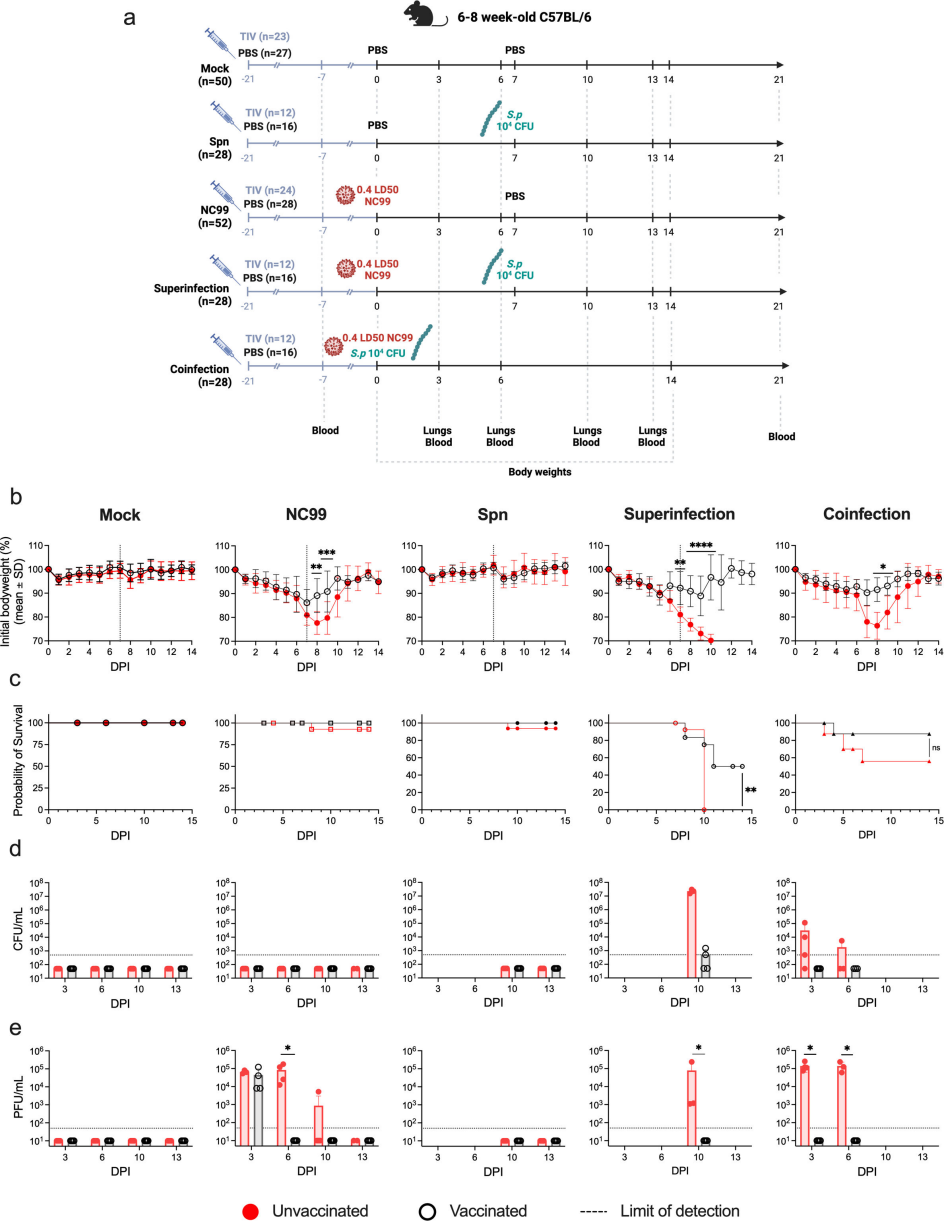
A single TIV vaccination has protective effects against secondary bacterial infections. (**a**) Schematic and graphical representation of experimental timeline. Visual representation of the five conditions: Mock, NC99 infected, Spn infected, superinfection, and coinfection. The timeline highlights days of treatment, infection, and end-point events for sample extraction. Created with: biorender.com (**b–e**) Panels showing mice bodyweight, survival curves, and lung bacterial and viral titers for the different experimental groups. Female C57BL/6 were vaccinated with TIV (black) or mock vaccinated (red) intranasally inoculated with 50 µL of PBS, 0.4LD_50_ of NC99, 10^4^ CFU of Spn, or a combination of infections (coinfection and superinfection). Graphical presentation displaying weight loss (**b**), mortality (**c**), bacterial (**d**), and viral (**e**) titers in the lungs of mice under various conditions. Data from three independent experiments were combined. Differences in bodyweight, bacterial and viral titers assessed by Mann-Whitney *U* test and differences in survival were assessed by Log Rank (Mantel-Cox) test. Significance values are represented as *P ≤ 0.05, **P ≤ 0.01, ***P ≤ 0.001, ****P ≤ 0.0001.

First, we investigated the potential differences in disease outcome between Spn coinfections and superinfections and evaluated if TIV could have a protective role against Spn secondary infections (Fig. 1). We established a C57BL/6 mouse model that was vaccinated with a single dose of TIV [equivalent of 3 µg HA, one-fifth of the human dose] or mock vaccinated with PBS, a similar model to the one we previously established for Sa (38). This vaccination regime is suboptimal and conducive to influenza breakthrough infections that cause reduced morbidity [approximately 10% bodyweight loss at 7 days post-infection (DPI)] compared to unvaccinated animals (approximately 20% bodyweight loss), providing a model of co- and superinfection with pre-existing influenza virus-specific immunity. Twenty-one days after vaccination, mice were intranasally challenged with either 50 µL of PBS, a sublethal dose of NC99 (632 plaque forming units [PFU], equivalent to 0.4 lethal dose 50% [LD_50_]), a sublethal dose of Spn (10^4^ colony forming units [CFU]), or both (superinfection and coinfection). For coinfection, bacterial and viral intranasal challenges were performed simultaneously on the day of infection (0 DPI), whereas for superinfection, mice were challenged with NC99 at 0 DPI and with Spn at 7 DPI (Fig. 1a).

As expected, initial infection with NC99 alone induced acute, self-limiting disease, with high morbidity, as reflected by a mean bodyweight loss at 7 DPI of 19.02%. In contrast, Spn infection alone caused no significant morbidity (Fig. 1b). While the morbidity associated with coinfection was only slightly higher than NC99 alone (22.03% bodyweight loss at 7DPI), survival rates for coinfected mice decreased considerably compared to NC99 alone (56% survival and 92.5% survival, respectively). Superinfection proved to be more lethal than coinfection with every mouse reaching humane endpoint by 10 DPI, only 3 days after exposure to Spn (Fig. 1c). Detectable lung bacterial titers were only found in co- and superinfected animals, but not in single Spn infections. Superinfection led to higher lung bacterial titers 3 days after bacterial challenge compared to coinfection (2.29 × 10^7^ CFUs/mL for superinfection and 3.13 × 10^4^ CFUs/mL for coinfection) (Fig. 1d). Lung viral titers presented no significant differences between bacterial coinfection or NC99 alone at 3 or 6 DPI. At 10 DPI, while virus titers were cleared in 5 of 6 animals infected only with NC99, the superinfected group still presented high titers (7.91 × 10^4^ PFUs/mL), underscoring the impairment of efficient antiviral immune responses in the context of a Spn superinfection (Fig. 1e).

After vaccination, the mice morbidity and mortality were significantly reduced. Vaccinated mice challenged with NC99 alone lost significantly less bodyweight and started recovering sooner than unvaccinated mice. Similar observations were made in coinfected animals, for which vaccination also improved survival (from 56% to 87.5%). Vaccination also reduced superinfection morbidity and mortality, significantly reducing body weight loss after Spn challenge and improving survival from 0% to 50% (Fig. 1b and c). Moreover, lung bacterial growth was controlled as soon as 3 DPI in coinfected mice, and only half of the superinfected mice presented detectable titers at 10 DPI (Fig. 1d). In line with previous findings for our challenge model, some NC99 breakthrough infection is observed, with animals challenged with NC99 alone presenting comparable lung viral titers at 3DPI in vaccinated and unvaccinated animals (4.4 × 10^4^ PFUs/mL and 6.5 × 10^4^ PFUs/mL, respectively) but controlling the infection much faster, with no detectable titers at 6DPI. Strikingly, vaccinated animals that were coinfected cleared viral infection more efficiently than those challenged with NC99 alone, showing no detectable titers already at 3 DPI. Lastly, vaccination resulted in no detectable lung viral or bacterial titers at 10 DPI in superinfected mice (Fig. 1e).

### Increased expression of Siglec-F in lung neutrophils and eosinophils correlates with protection from bacterial secondary infections after TIV vaccination

**Fig 2:**
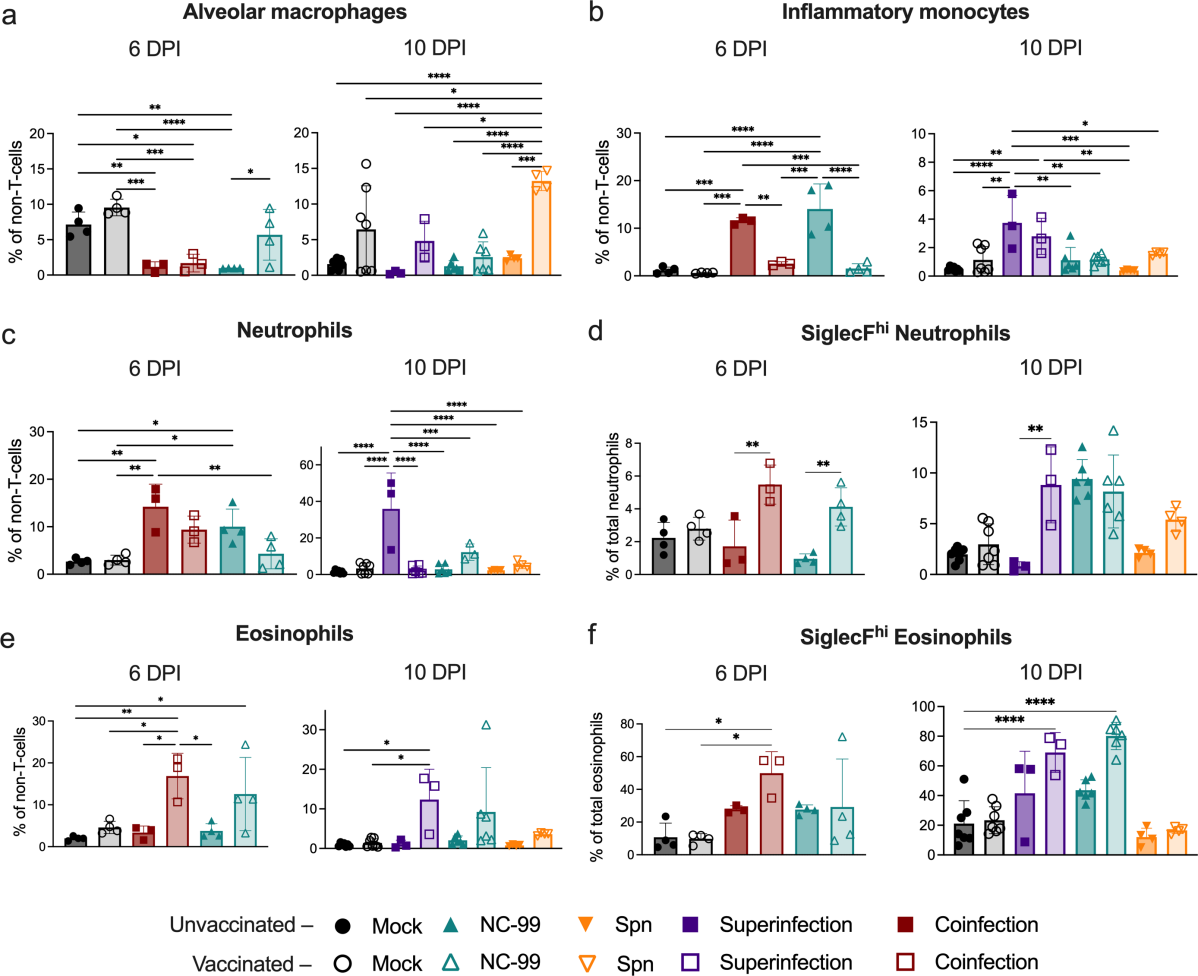
TIV vaccination prevents from exacerbated lung neutrophil recruitment and promotes eosinophil recruitment. Relative quantification of cells identified in lung by flow cytometry. Frequency of (**a**) alveolar macrophages, (**b**) inflammatory monocytes, (**c**) neutrophils, (**d**) SiglecF^hi^ neutrophils, (**e**) eosinophils, and (**f**) SiglecF^hi^ eosinophils in lungs at 6 and 10 DPI. Data from three independent experiments were combined. Comparisons were performed by one-way ANOVA with Tukey’s multiple comparisons test. Significant values are represented as *P ≤ 0.05, **P ≤ 0.01, ***P ≤ 0.001, ****P ≤ 0.0001.

To further characterize the immune response after infection with and without vaccination in the different conditions, lungs were collected on 6 and 10 DPI. Lungs were dissociated into a single cell suspension, and relative quantification of immune cell subsets was performed by flow cytometry (Fig. 2; Fig. S1). Gating strategy is detailed in Fig. S2. Alveolar macrophage dysregulation has been characterized as one of the key mechanisms triggered by influenza infection that promotes increased susceptibility to bacterial secondary infections (33). Alveolar macrophages exhibited a significant reduction in NC99-challenged mice at 6 DPI, which TIV vaccination largely prevented. Interestingly, coinfected animals showed a similar reduction in alveolar macrophages at this stage; however, there was no significant protection of these cells provided by vaccination (Fig. 2a). Infiltration of inflammatory monocytes was significantly increased in both coinfection and NC99 alone groups, but it was prevented by vaccination in both groups (Fig. 2b). At 10 DPI, alveolar macrophages remained low in unvaccinated mice challenged with NC99 alone, similar to superinfected animals, while vaccination showed a trend of alveolar macrophage protection in both groups (Fig. 2a). Protection from alveolar macrophage loss by TIV vaccination was even detected in Spn-only challenged animals although further investigation is required to characterize this phenomenon. Interestingly, inflammatory monocytes presented elevated levels in all superinfected animals, regardless of their vaccination status, and significantly higher than the ones in animals infected with NC99 alone or Spn alone (Fig. 2b).

Exacerbated lung neutrophilic inflammation in influenza virus-Spn coinfections has been associated with pneumococcal persistence in the lung and increased disease severity (42). At 6 DPI, we observed a significant increase in neutrophil infiltration in NC99-challenged unvaccinated mice (10.02% ± 3.69%) and coinfected mice (14.21% ± 4.75%) compared to the mock unvaccinated condition (2.66% ± 0.65%). A non-significant trend toward the reduction of neutrophilic infiltration by vaccination was observed in mice challenged with NC99 (4.30% ± 3.16%) and coinfected (9.38% ± 2.86%). At 10 DPI, a very strong neutrophil recruitment was observed in unvaccinated superinfected mice, reaching mean values of 35.93% ± 19.64% of total non-T-cells, compared to 1.35% ± 0.97% from unvaccinated mock. Vaccination achieved significant control of neutrophilic infiltration although neutrophil levels were still elevated (12.22% ± 4.50%) compared to mock or NC99 alone (Fig. 2c). Lung neutrophil subpopulations are still poorly characterized, but they can be assessed based on their expression of Siglec-F. While we observed that neutrophilic lung infiltration was clearly reduced by vaccination, as outlined above (Fig. 2c), the percentage of Siglec-F^hi^ activated neutrophils increased after vaccination in all infected groups. At 6 DPI, both coinfected and NC99 challenged groups that received the vaccine showed a significant increase when compared to the unvaccinated counterparts. Interestingly, at 10 DPI, while TIV vaccination still increased the percentage of Siglec-F^hi^ neutrophils in all infected groups, unvaccinated mice receiving NC99 alone also showed an elevated percentage, similar to the mice that received the vaccine (Fig. 2d).

The role of eosinophils in viral infections, especially in the context of vaccination, remains a highly debated topic (43), with both examples of pathological effects mostly associated with vaccine-associated enhanced respiratory disease (VAERD) (44) and protective effects in the context of breakthrough respiratory infections (45, 46). In our model, an increase in lung eosinophil recruitment was observed for vaccinated groups challenged with NC99 alone (both at 6 and 10 DPI), coinfected (6 DPI), and superinfected (10 DPI), but was absent in all the groups that did not receive the vaccine (Fig. 2e). Similar to neutrophils, lung eosinophils can also be classified based on Siglec-F expression, with Siglec-F^hi^ eosinophils corresponding to the inflammatory eosinophil subset and Siglec-F^int^ eosinophils corresponding to tissue-resident eosinophils. Functionally, Siglec-F^hi^ eosinophils have been associated with exacerbated Th2 responses in murine models for asthma (47, 48). However, previous results from our group and others suggest a protective role of these Siglec-F^hi^ eosinophils in the context of influenza (45, 49). Opposite to the total neutrophil population, total eosinophils increased in vaccinated groups challenged with NC99 alone, coinfected, or superinfected, compared to unvaccinated. In line with our previous findings, the percentage of eosinophils classified as Siglec-F^hi^ significantly augmented in vaccinated animals that were coinfected (6 DPI) and challenged with NC99 alone or superinfected (10 DPI). Unvaccinated mice challenged with NC99 also show an increase in inflammatory eosinophils compared to the mock although milder than the one in vaccinated groups (Fig. 2f).

Lung CD3^+^ cells did not reveal clear differences among experimental groups. Dendritic cells in the lung increased in vaccinated animals when compared to unvaccinated groups at both 6 DPI and 10 DPI (Fig. S1).

### Cytokine and chemokine profiling of the lungs of superinfected infected mice reveals a differential inflammatory profile

**Fig 3:**
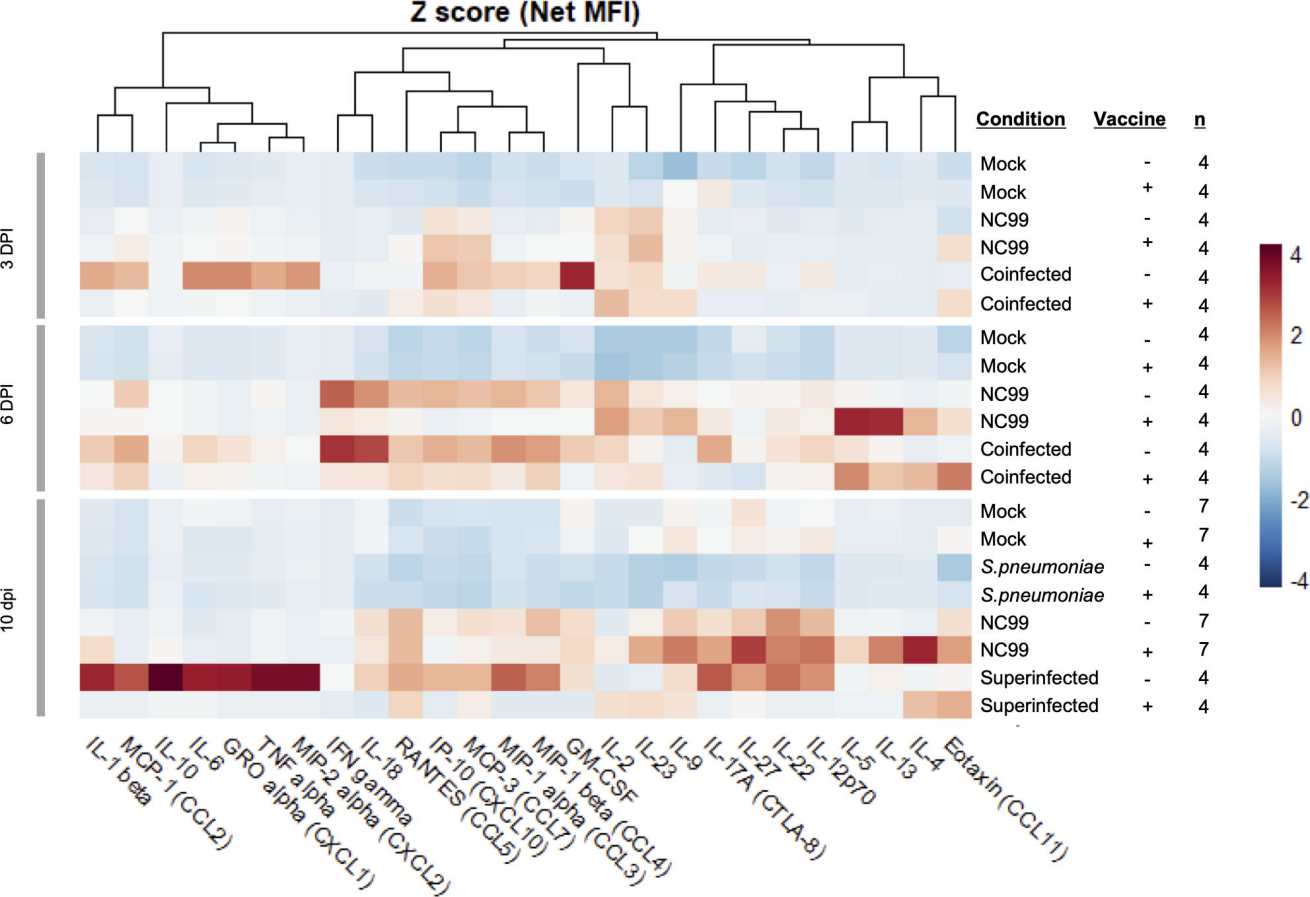
TIV vaccination reduces pro-inflammatory cytokine and chemokine production in lungs from coinfected and superinfected animals. Cytokine profiling from mice lungs at 3, 6, or 10 DPI by Luminex multiplex cytokine ELISA. Data from three independent experiments were combined. Heatmap of cytokine and chemokine Z-scored Average Net MFI.

To study cytokines and chemokines associated with the phenotype of the different infection regimes in the lungs of the infected mice, we performed a multiplex-ELISA (Luminex) quantification of lung homogenates supernatants at 3, 6, and 10 DPI (Fig. 3, Fig. S3 to S5). At 3 DPI, mock and Spn infected mice, regardless of their vaccination status, did not show increased levels of any of the cytokines and chemokines measured. Unvaccinated mice infected with NC99 showed non-significant increases in the pro-inflammatory chemokines CCL5, CCL7, and CXCL10 compared to mock. Those chemokines were even more elevated in NC99-challenged vaccinated mice. Coinfected animals that did not receive TIV presented a further exacerbated proinflammatory profile, with significantly higher levels of CCL5, CCL7, CXCL10, and IFN-γ. IL-2 was also elevated in all groups challenged with NC99 or coinfected, regardless of their vaccination status. Eosinophil-recruiting eotaxin was significantly elevated in vaccinated groups challenged with NC99 alone or coinfected, in the absence of any of the classical type-2 skewing cytokines (IL-4, IL-5, or IL-13).

At 6 DPI, differences between vaccinated and unvaccinated mice challenged with NC99 became more apparent, in accordance with the self-limiting nature of the breakthrough infections in the model described above. Chemokines CCL5, CCL7, and CXCL10 remained significantly elevated in unvaccinated mice, while they returned to mock levels or were reduced in vaccinated mice. Unvaccinated coinfected mice presented a more inflammatory cytokine profile at 6 DPI than at 3 DPI, with significantly elevated levels of cytokines IL-6, IFN-γ, and IL-18, and chemokines CXCL1, CXCL2, CXCL10, CCL3, CCL4, and CCL7. All these cytokines and chemokines were at the base level or reduced in TIV-vaccinated mice. IL-2 remained elevated in all groups challenged with NC99 or coinfected, regardless of their vaccination status. Eotaxin was not significantly elevated anymore by 6 DPI in vaccinated animals challenged with NC99 or coinfected although non-significant increases in anti-inflammatory cytokines IL-4 and IL-13 could be observed in lungs of vaccinated NC99-infected and coinfected mice at 6 DPI.

Superinfection generated a divergent chemokine and cytokine profile in the lung at 10 DPI when compared to animals challenged only with NC99. While chemokines such as CCL2, CCL7, and CXCL10 and cytokines such as IL-2 or IFN-γ were still elevated, they trended downward in NC99 challenged mice, but they were further increased in superinfected animals. Additionally, other cytokines, such as IL-6 and IL-10, and chemokines CXCL1 and CXCL2 were highly elevated in the superinfected group. TIV vaccination completely abrogated any increase for most of those cytokines and chemokines, further highlighting the protective role of TIV not only against bacterial proliferation, but also against inflammation.

### A single TIV vaccine dose efficiently induces neutralizing antibodies against NC99, which are boosted by infection, regardless of the secondary bacterial infection status

**Fig 4:**
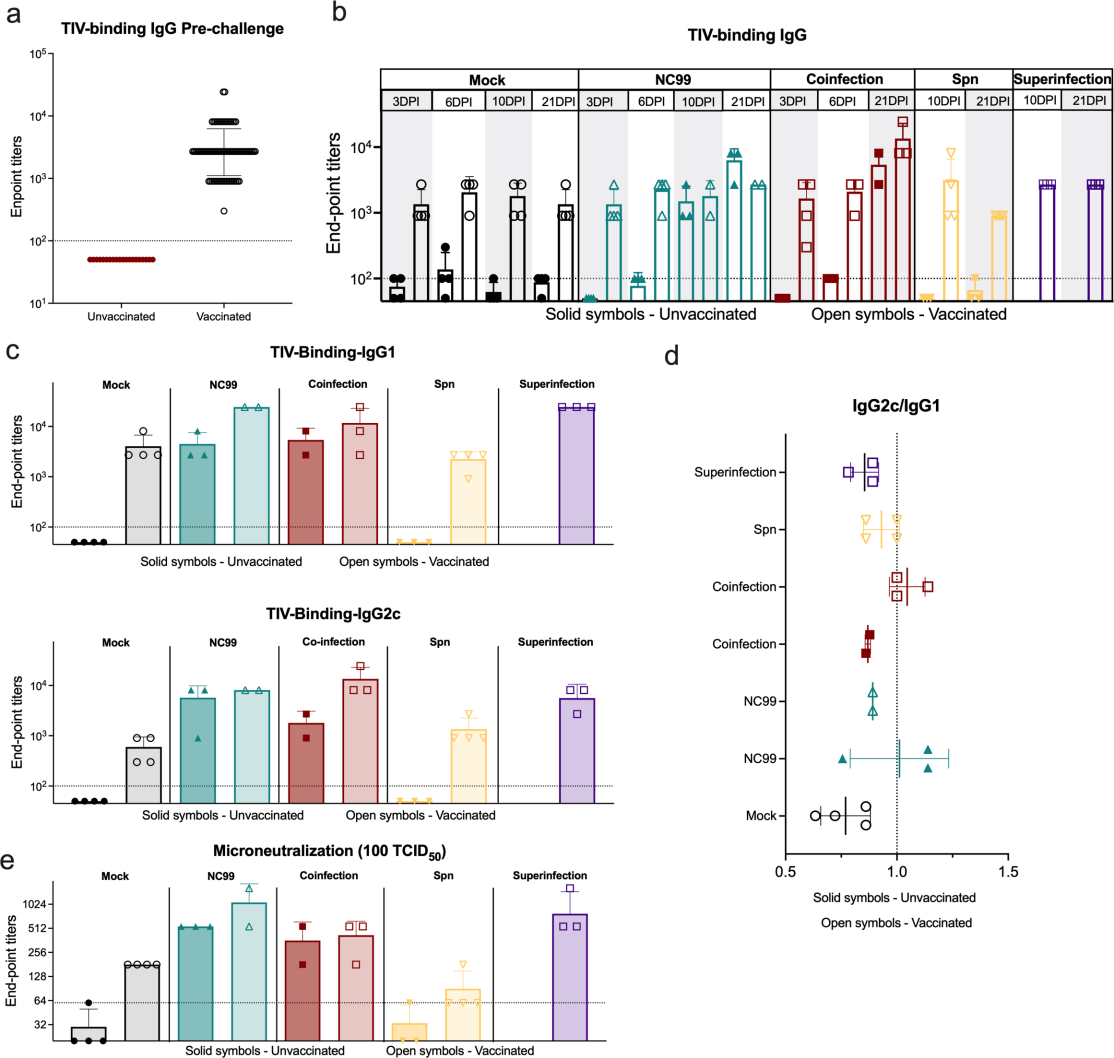
TIV vaccination induces neutralizing antibodies against NC99 with superinfection and coinfection skewing antibody responses. (**a**) TIV-binding IgG endpoint titers 2 weeks after a single-dose TIV vaccination. (**b**) Kinetics of TIV-binding IgG after challenge, at 3, 6, 10, and 21 DPI. (**c**) TIV-binding IgG1 and IgG2c 21 DPI. (**d**) Coefficient of the log10 of the endpoint titers of TIV-binding IgG2c over the log10 of the endpoint titers of TIV-binding IgG1 (indicative of Th1/Th2 antibody skewing). (**e**) Endpoint titers of the microneutralization assay against 100 TCID_50_ of NC99 in MDCK cells. Data from three independent experiments were combined.

To evaluate the role of Spn coinfection and superinfection in the humoral response after influenza infection and vaccination, half of the mice included in this study were vaccinated with a single dose of TIV vaccine, and the other half of the mice received a control PBS injection. Two weeks later, TIV-binding IgG titers were measured, and all vaccinated animals showed positive seroconversion (Fig. 4a). The kinetics of the antibody response were also evaluated after the challenge. Given the setup of this experiment with many mice at the beginning, mock-challenged unvaccinated animals showed no detectable TIV-binding IgG, while mock-challenged vaccinated animals showed detectable TIV-binding IgG titers that remained constant from 3 DPI to 21 DPI. Comparable results were obtained in the group that was only challenged with Spn. Challenge with NC99 alone led to the production of detectable TIV-binding IgG titers as soon as 6 DPI in unvaccinated animals. By 21 DPI, vaccinated and unvaccinated animals challenged with NC99 showed comparable IgG titers. While Spn superinfection did not have a significant effect on TIV-binding IgG titers in vaccinated animals, coinfection led to a significant increase in TIV-binding IgG titers when compared to mock-challenged vaccinated animals (*P* < 0.0001), but more interestingly also compared to NC99-challenged vaccinated mice (*P* < 0.0001) (Fig. 4b), which correlates with the faster viral clearance observed in vaccinated coinfected mice. No detectable specific antibodies could be collected for the 21 DPI timepoint for unvaccinated animals in the superinfection group as all mice reached humane endpoint prior to that timepoint.

Then, we evaluated the effect of vaccination and secondary bacterial infection on IgG subclasses. Both IgG1 and IgG2c were boosted by infection 21 DPI in all groups (Fig. 4c). No significant differences were found between vaccinated mice challenged only with NC99 or coinfected with Spn. Vaccinated superinfected mice showed significantly higher levels of TIV-binding IgG1 compared to vaccinated coinfected mice (*P* = 0.0254) (Fig. 4c). Consequently, vaccinated superinfected mice showed a slightly more Th2-skewed antibody response when compared with vaccinated mice that were coinfected, which present a more balanced Th1/Th2 antibody response (Fig. 4d).

Finally, we performed microneutralization assays to evaluate neutralizing antibody responses against NC99. Vaccinated mice that were superinfected or infected with NC99 alone showed higher microneutralization endpoint titers than vaccinated animals that were coinfected; however, these differences were not significant (Fig. 4e).

In all, our results show that TIV vaccination has a major role in the protection against disease and mortality in influenza-infected mice due to secondary bacterial infections, reflected as well in the rate of viral and bacterial clearance, the cellular infiltration in the lung, the cytokine and chemokine profile, and the antibody immune response.

## DISCUSSION

Despite the numerous advances in this field, complications derived from Spn secondary bacterial infections in the context of influenza remain a global healthcare problem. Several studies have shed light on the mechanisms underlying the interplay between these two pathogens (17, 50–53). However, many questions remain unanswered. Comparative analyses evaluating secondary bacterial infections based on the timing of the bacterial challenge with respect to the beginning of the influenza infection are scarce. Still, relative timing of the two infections could play a major role in the outcome of the disease due to the differential kinetics of influenza and Spn infections within the host. Here, we distinguished between coinfection (simultaneous infection with both pathogens) and superinfection (infection with Spn 7 days after NC99 challenge), comparing, from an integrative point of view, protection from disease, bacterial and viral clearance, and cellular and humoral immune response to influenza virus in the two different scenarios. We also explored the role of pre-existing immunity to influenza, provided by TIV vaccination, on modulation of the host response to infection.

Our results show that mortality rates are elevated in both coinfections and superinfections in the context of NC99 and Spn challenges that are sublethal on their own. Relevant differences were detected between the two secondary infection regimes. Superinfection led to 100% mortality within 3 days of the bacterial challenge, while coinfection showed less than 50% mortality. Therefore, the timing of both infections determines the degree of synergism between bacteria and virus to cause a more lethal disease. Other researchers have reported similar observations, where infection with Spn 7 days before influenza infection did not affect the mortality, coinfection at 0 DPI decreased survival rates by almost 50% and if the bacterial infection occurred 3, 5, or 7 days after influenza challenge (superinfection), the lethality reached 100%. Infection with Spn at later timepoints was progressively less lethal (54). Notwithstanding, these results might be dependent on a plethora of factors, including genetic background of the pre-clinical model or bacterial and viral strains. For example, another study observed extreme susceptibility to Spn in superinfected C57BL/6, the model used in our study, but not in BALB/c (16).

Additionally, we investigated the impact of vaccination with TIV in our co- and superinfection model. Previously, our group has shown the protective capacity of this vaccine against superinfections with *Staphylococcus aureus* (38). Here, we show that even a single dose of TIV, which is considered a suboptimal vaccination regimen as it allows breakthrough influenza infections, significantly increased survival in both superinfections and coinfections, but with different efficacy. This effect was primarily explained by the viral and bacterial titers found in mouse lungs, with lower lung virus titers in vaccinated mice, compared to unvaccinated mice. In unvaccinated NC99 mice, viral clearance was almost complete by 10 DPI. However, unvaccinated superinfected mice showed prolonged virus replication with detectable virus even at 10 DPI. This delay in viral clearance has been previously described and correlated with the initial bacterial inoculum size, among other factors (13). These results align with what we observed in our experiments and emphasize the beneficial effects of vaccination in secondary bacterial infections. This is further highlighted by the results obtained in coinfected animals. A single TIV dose is not sufficient to provide sterilizing immunity in mice, as we have previously established in our pre-clinical models (38, 45, 49). Here, while breakthrough infection was detectable 3 DPI in vaccinated mice that were challenged only with NC99, coinfected mice were able to clear the virus more efficiently and showed no breakthrough infection at 3 DPI. While the literature describing the effects of influenza infections in the clearance of bacterial secondary infections is abundant, the effect of prior or concurrent bacterial infections in viral clearance is less characterized. Early viral control due to prior bacterial infections has been described in other bacteria-virus combinations, such as SARS-CoV-2 and *Mycobacterium tuberculosis*. This control took place as early as 1 day after viral challenge and was dependent on bacterial dose. The authors speculate that *Mycobacterium*-driven innate inflammation (through PPRs signaling) together with the induction of antiviral interferons may mediate this early protection; however, they are not able to isolate a specific signaling pathway or combination of them responsible for this response (55). The impact of co-administration of bacteria and virus on the host innate immune response, eventually resulting in better virus control, requires further study.

According to literature, mice that survive Spn infection usually reach bacterial clearance within the third day after infection. On the other hand, if bacterial disease manages to progress, mice typically die between the second and the fourth day after bacterial infection (54). In line with these observations, in our model, unvaccinated mice infected with only Spn did not present detectable bacteria in lungs at 3 DPI, while in coinfected animals, Spn was found even at 6 DPI.

It is well established that influenza infection decreases macrophages’ phagocytic ability (13), as well as the absolute number of alveolar macrophages as soon as 3 DPI (50, 56). During the initial week post-influenza infection, over 90% of resident alveolar macrophages are depleted, with the surviving cells exhibiting a necrotic phenotype (50). After the depletion of alveolar macrophages upon influenza infection, they are repopulated with bone-marrow monocyte-derived alveolar macrophages within days 7–11 (56). These results are in accordance with the extensive macrophage depletion we observed that in both coinfection and superinfection models, that is partially avoided by TIV vaccination. Therefore, a possible explanation for the enhanced mortality if Spn infection occurs after influenza virus infection is due to the loss of alveolar macrophages that occurs in the days post influenza virus infection (49); since alveolar macrophages are a first line of defense against bacterial infections, virus-mediated macrophage death may leave mice more susceptible to severe disease outcome during superinfection.

The secondary stimulus for superinfection triggered a massive recruitment of neutrophils. Exacerbated lung neutrophilic infiltration has been widely characterized in superinfection (57), and it is commonly related to functional impairment in the lungs and lethality. Both coinfected and superinfected mice showed an increase in neutrophil recruitment, that is prevented with a single vaccination dose of TIV. This is paired with the cytokine profile of both conditions, exhibiting elevated values of IL-6, CXCL1, and CXCL2, which induce the production and recruitment of neutrophils. Some studies have focused on neutrophils in superinfections as a major cause of lethality and found protective effects of CXCR1/2 antagonist during Spn superinfections (58). Once again, TIV vaccination largely prevented excessive neutrophil recruitment to the lung. We further characterized neutrophil populations in the lung based on their expression of Siglec-F. Dogmatically, Siglec-F expression was typically attributed to eosinophils and alveolar macrophages in the mouse lung. However, a Siglec-F^hi^ lung neutrophil subset has recently been described in the allergy field, characterized by a higher activation and increased effector functions such as neutrophil extracellular trap (NET) release and airway hyperresponsiveness (59, 60). Siglec-F^hi^ neutrophils have also been associated with protection against nasal challenge with *Bordetella pertussis*. Mobilization of this neutrophil subset was mediated by IL-17 and was lost, together with the improved protection, in IL17A^−/−^ mice (61). In our model, while exacerbated neutrophil recruitment is greatly prevented in TIV vaccinated groups, an increased fraction of Siglec-F^hi^ neutrophils is detected when compared to unvaccinated controls. Therefore, this neutrophil subset could have a role in the observed improved protection against bacterial secondary infections in those groups. Nevertheless, IL-17A concentration in lung supernatants shows very limited differences among groups. Thus, further mechanistic studies are needed to define how these neutrophils are recruited in the context of vaccination and what their role in protection.

A pronounced lung eosinophilic recruitment was also detected in vaccinated mice challenged with NC99 alone or co/superinfected. Pulmonary eosinophilia is typically associated with negative outcomes of infection in vaccinated individuals, in a phenomenon known as vaccine-associated enhancement of respiratory disease (VAERD), initially described in the context of respiratory syncytial virus vaccination (62, 63). However, growing scientific evidence also suggests an antiviral role for eosinophils in both mice and humans (64, 65). Our group has previously described similar findings, with vaccine-associated pulmonary infiltration of eosinophils in the influenza mouse and hamster models that rather correlates with protection in the absence of VAERD, in the context of SARS-CoV-2, influenza infection, and superinfection with Sa. This pulmonary eosinophilia is vaccine-dose dependent and is present regardless of the immune skewing of the host caused by the vaccine adjuvant (38, 43, 45, 46).

While we show that a single dose of TIV vaccine is enough to provide detectable TIV-binding IgG titers, virus-specific antibody titers are boosted differently after infection. Notably, vaccinated animals that were coinfected with Spn show significantly higher titers at 21 DPI than animals that received TIV and that were challenged with NC99 alone. Comparatively, superinfected mice, even in the context of vaccination, showed reduced antibody titers compared to coinfected animals. A reduced B-cell response against influenza virus during superinfection is well characterized in the literature, with superinfected mice showing reduced levels of virus-specific IgG, IgM, and IgA, as well as the number of B cells, CD4^+^ T cells, and plasma cells (66). Superinfections have also been associated with spleen atrophy resulting in massive splenic B lymphocyte apoptosis through the mitochondrial pathway when compared to influenza infection alone (16). However, these studies have been performed only in the context of superinfection, where alveolar macrophages are already depleted by virus infection. Coinfected animals showed the strongest Th1 skewed humoral response, which might be helping in the early viral clearance detected at 3DPI. In human clinical trials, while nasal pneumococcal colonization had no impact upon TIV-induced antibody responses in serum, pneumococcal colonization dampened vaccine-mediated mucosal antibody responses, measured as Influenza-binding IgA and IgG in nasal washes (67). These results warrant further investigation of mucosal immune responses after challenge in our models.

Overall, our results highlight the importance of influenza vaccination in the prevention of severe disease following bacterial secondary infections. They also underline important differential outcomes of the antiviral host response during coinfections and superinfection and their divergent effects in antibody response to TIV vaccination. We show that both coinfection and superinfection cause neutrophilic inflammation in the lung that correlates with detrimental outcomes and is partially prevented by vaccination. However, SiglecF^hi^ neutrophils are increased in vaccinated animals and may play a protective role. More mechanistic studies are needed to understand the underlying immune mechanisms that drive these observations. Finally, we observed lung eosinophilia that correlated with protection from coinfection and superinfection in vaccinated animals, thereby confirming observations reported before by our research group for the influenza vaccination/challenge model.

## MATERIALS AND METHODS

### Cells

MDCK cells (female origin Madin-Darby canine kidney cells, ATCC CRL-3216) were used to determine the NC99 titration. The cell lines were maintained in Dulbecco’s modified Eagle’s medium (DMEM, GIBCO) supplemented with 10% (vol/vol) fetal bovine serum (GIBCO) and 1× penicillin/streptomycin at 37°C under a humidified atmosphere containing 5% CO_2_.

### Bacteria

*Streptococcus pneumoniae* ATCC6301 (serotype 1) was grown and maintained at 37 °C, 5% CO2 on Columbia blood agar plates (Thermo Fisher). Overnight cultures were prepared by adding a single colony in 40 mL of brain heart infusion (BHI) broth (Sigma). Then, 3 mL of grown culture was transferred to a new tube with 12 mL of fresh BHI media and incubated between 4 and 6 h until it reached an Optical Density of 600 nm (OD600) equal to 1. The number of bacteria in the final suspension was determined by plating 10-fold serial dilutions onto agar plates. Titration of bacteria showed that an OD600 approximately had 2.665 × 10^8^ CFU/mL. Then, 1 mL was centrifuged at 2,000 rpms for 5 min. The supernatant was discarded, and the pellet was resuspended in 1 mL of PBS and diluted to obtain the desired number of bacteria for infection. Bacteria were identified as pneumococci by alpha-hemolysis on blood agar. Storage of bacteria was performed at mid-exponential phase (5–6 h) in BHI, with the addition of 10% glycerol, and storage at −80 °C. To enhance bacterial specificity to murine hosts, strains were passaged once within murine lungs, subsequently recovered, and cryopreserved according to the previously described protocol.

### Virus

The virus used for challenge was H1N1 A/New Caledonia/20/1999 (NC99) at a sublethal dose (0.4 LD_50_, 632.4 PFU/mL). NC99 was contained in the formulation of the TIV vaccine used for immunization

### Vaccine

For all vaccination experiments 1/5 of the human dose of the seasonal trivalent inactivated influenza virus vaccine was administered intramuscularly in the hind legs (TIV; Fluzone Influenza Virus Vaccine) containing an influenza A H1N1 component (A/New Caledonia/20/1999/IVR-116), influenza A H3N2 component (A/New York/55/2004/X-157 [an A/California/7/2004-like strain]), and influenza B component (B/Jiangsu/10/2003 [a B/Shanghai/361/2002-like strain]).

### Mouse infection models

All experiments were performed with 6- to 8-week-old female C57BL/6 mice (Strain #000664, The Jackson Laboratory).

A standardized suspension of the ATCC6301 strain was prepared for inoculation by overnight growth in BHI from a single colony. The next day, subculture was performed, and the bacteria were allowed to grow to mid-exponential phase (5–6 h). To standardize the initial dose of infection, optical density (OD600) was measured at different timepoints until the absorbance at 600 nm reached values of 1. Then, bacteria were harvested by centrifugation and suspended in phosphate-buffered saline (PBS). This suspension was diluted 1/10 and titrated in Columbia blood agar. For the initial infection, 1,000 CFUs of ATCC6301 strain were used according to titrated values at OD600 = 1. For the viral challenge, 0.4 LD_50_ of NC99 was diluted in PBS to the appropriate infection volume.

Mice were intranasally challenged under mild ketamine/xylazine anesthesia. A final infection volume of 50 μL was used for all experiments. If coinfection was performed, bacteria and virus were combined in a single suspension administered in the same final volume. A detailed experimental layout is presented in Fig. 1a.

### Bacterial titatrion by Miles & Misra quantification

The upper lobe from the right lung of the mice was collected in 500 µL of 1× PBS. On the same day of the assay, lungs were disrupted using a cell strainer (70 µm) and the back of a sterile syringe. Homogenates were collected in 1.5 mL Eppendorf tubes. Serial 1/10 dilutions of each lung were performed in 96-well plates. Dilutions were plated by adding a single drop of 20 µL on Columbia blood agar plates according to Miles & Misra titration method (68). Technical triplicates of each lung were performed. Agar plates were incubated overnight in an incubator at 37°C with 5% CO_2_. The next day, single colonies were counted to determine lung bacterial titers.

### Virus titration by plaque assays

NC99 titration was performed as previously described (45). Briefly, the middle lobe from the right lung was collected in 500 µL of PBS in prefilled homogenizer bead tubes containing 3.0 mm high impact zirconium beads (Benchmark Scientific), snap-frozen on dry ice after harvest and stored at −80°C. On the day of the assay, samples were thawed, homogenized, and then centrifuged at 5,000 × *g* for 5 min at 4°C. Supernatants were 10-fold serially diluted in PBS from 1/10 to 1/10^6^. 12-well tissue culture plates were seeded the day prior with 3.5 × 10^5^ MDCK cells. The day of the assay, plates were washed once with 1 mL of PBS per well. After washing, 250 µL of each lung supernatant dilution was added to the plate and then incubated for 1 h at room temperature (RT). After incubation, cells were washed once with 1 mL/well of PBS, and then 1 mL/well of overlay was added to each well. The overlay solidified at RT for 15 min and then plates were incubated at 37°C, 5% CO_2_ for 48 h. Then, plates were fixed with 1 mL of 4% formalin per well O/N at 4°C. After washing with PBS-T (PBS + 0.05% Tween 20), monolayers were immunostained with 200 μL/well polyclonal mouse serum diluted in blocking buffer (5% milk in PBS) for 1.5 h on a plate rocker. Plates were washed one time with PBS-T then 200 μL/well of horseradish peroxidase (HRP)-conjugated goat anti-mouse IgG Fc secondary antibody diluted in blocking buffer for 1 h at room temperature while rocking. Plates were washed, and then 150 μL/well KPL TrueBlue was added and incubated at room temperature for 30 min on a plate rocker. Plates were washed, and plaques were counted to determine lung viral titers.

### Flow cytometry

To analyze the lung cell populations at 6 and 10 DPI, the left lung lobe was collected and made into a single cell suspension using a Mouse Lung Dissociation Kit (Miltenyi Biotec), following manufacturer instructions. In brief, gentleMACS C tubes were filled with 2.4 mL of Buffer S and kept on ice while lungs were harvested. Then, each tube was spiked with 100 µL of Enzyme D and 15 µL of Enzyme A. Then, C Tubes were attached it upside down onto the sleeve of the gentleMACS dissociator, where they were incubated at 37°C during 32 min, following the 37C_m_LDK_1 program of the gentleMACS dissociator.

After generating a single cell suspension, cells were centrifuged for 5 min at 500 × *g,* and the supernatant was decanted. Then, cells were resuspended into 2 mL of RBC lysis solution (5 PRIME) and incubated at RT for 10 min. Then, 10 mL of PBS was added to stop lysis, and cells were centrifuged for 5 min at 500 × *g,* and the supernatant was decanted. Then, cells were resuspended into 50 μL of Fc Block and incubated for 5 min at room temperature. Meanwhile, a staining cocktail solution was prepared diluting 1 μL of BD Pharmingen FITC Hamster Anti Mouse CD3e (Clone 145-2C11), BD Pharmingen PE Rat Anti-CD11b (Clone M1/70), BD Horizon PE-CF594 Rat Anti-Mouse Siglec-F (Clone E50-2440), BD Pharmingen PE-Cy7 Hamster Anti-Mouse CD11c (Clone HL3), Ly-6C Monoclonal Antibody, APC, eBioscience, Invitrogen (Clone HK1.4), Alexa Fluor 700 anti-mouse Ly-6G Antibody (Clone 1A8), and 0.5 μL of Fixable Viability-e780 and MHC Class II (I-A/I-E) Monoclonal Antibody, eFluor 450, eBioscience (Clone M5/114.15.2) into a final volume of 50 μL in FACS buffer. The staining cocktail was added on top of the samples, and they were incubated for 20 min at room temperature protected from the light. After the incubation period, cells were centrifuged for 5 min at 500 × *g* and washed twice with 200 μL of FACS buffer. Samples were acquired using a Beckman Coulter Gallios flow cytometer with Kaluza software. Data analysis was performed using FlowJo v10.10 (Treestar) and compensated using the built-in AutoSpill algorithm. Data were visualized using GraphPad Prism v10.1.1.

### Lung cytokine and chemokine profiling

The middle lobe from the right lung of the mice was collected, homogenized, and clarified as described above. The Cytokine & Chemokine 26-Plex Mouse ProcartaPlex Panel 1 (ThermoFisher) was used to measure cytokine and chemokine concentrations in the lungs, following manufacturer’s instructions. In brief, 50 µL of lung homogenate was combined with beads in a flat-bottom black 96-well plate and incubated for 30 min at RT protected from light. All RT incubations were performed in a shaker at 300 rpm. After 30 min, the plate was moved to 4°C for overnight incubation. The next day, the plate was equilibrated to room temperature for 30 ms and then washed three times with 150 µL/well of Wash Buffer. Following washing, 25 µL/well of Detection Antibody mixture was added and incubated at room temperature for 30 min. The plate was washed three times, and then, 50 µL/well of Streptavidin-PE solution was added and incubated for 30 min at RT. After washing the plate three times, 120 µL/well of Reading Buffer was added and the plate was incubated for 5 min at room temperature. Data were acquired on a Luminex 100/200 analyzer (Millipore) with xPONENT software [version (v) 4.3]. Data visualization and analysis were conducted using GraphPad Prism (v10.1.1) and RStudio (v2022.12.0+353).

### Enzyme-linked immunosorbent assay

Mice sera were tested for vaccine-specific IgG, IgG1, and IgG2c titers. ELISA plates (Nunc MAXISORP, ThermoFisher) were coated with TIV vaccine diluted 1:100 into ELISA coating buffer (0.2M bicarbonate buffer, pH 9.6) and incubated O/N at 4°C. Plates were washed three times with washing buffer (1× PBS + 0.1% Tween20 [Sigma Aldrich]) and incubated in blocking buffer for 1 h at room temperature. The serum samples were threefold serially diluted starting with 1:100 dilution in blocking buffer and allowed to bind to the TIV-coated ELISA plates for 1 h 30 min. Then, the plates were washed three times with washing buffer and incubated with 1/5,000 diluted secondary total IgG (Sigma Aldrich) or 1/4,000 IgG1 or IgG2c antibodies (Southern Biotech), conjugated to horse radish peroxidase (HRP) for 1 h at room temperature. Finally, the plates were washed three times in washing buffer and incubated with 50 µL of tetramethylbenzidine substrate (TMB, BD) until blue color appeared. The colorimetric reaction was stopped by adding 50 µL of ELISA stop solution (ThermoFisher), and absorbance was measured at 450 nm and 650 nm wavelengths.

### Statistics

Pairwise comparisons were made by Mann-Whitney *U* test. Differences among three or more groups were performed by one-way ANOVA with Tukey’s multiple comparisons test and survival was assessed by Log Rank (Mantel-Cox) test. Statistical test is specified in each case in the figure legend. Significance values are represented as *P ≤ 0.05, **P ≤ 0.01, ***P ≤ 0.001, ****P ≤ 0.0001. GraphPad Prism (v10.1.1) and RStudio (v2022.12.0 + 353) were used for analysis and visualizations.

## Data Availability

The raw data supporting the conclusions of this article will be made available by the authors, without undue reservation.

## SUPPLEMENTAL MATERIAL

Supplemental material jvi.01774-25-s0001.pdf Figures S1 to S5.

ASM does not own the copyrights to Supplemental Material that may be linked to, or accessed through, an article. The authors have granted ASM a non-exclusive, world-wide license to publish the Supplemental Material files. Please contact the corresponding author directly for reuse.
