# Supplementary material for: Disease modulation by TIV vaccination during secondary pneumococcal infections in influenza-infected mice
Source: J Virol. 2025 Dec 29;100(2):e01774-25. doi: 10.1128/jvi.01774-25 (PMC12911902; doi:10.1128/jvi.01774-25)
Supplement: Supplemental material — Figures S1 to S5. [file jvi.01774-25-s0001.pdf]

TITLE

Disease modulation by TIV vaccination during secondary pneumococcal infections in influenza-infected mice.

AUTHORS

Juan García-Bernalt Diego[1,2,*], Javier Arranz-Herrero[3,4,5,6,*], Gabriel Laghlali[1,2,7], Eleanor Burgess[1,2,8], Seok-Chan Park[1,2], Gagandeep Singh[1,2], Lauren A. Chang[1,2,8], Prajakta Warang[1,2], Moataz Noureddine[1,2,8], Jordi Ochando[9,10], Estanislao Nistal-Villan[5,6,#] and Michael Schotsaert[1,2,11,12,#]

*These two authors contributed equally

#Correspondence to: estanislao.nistalvillan@ceu.es and michael.schotsaert@mssm.edu

AFFILIATIONS

[1]Department of Microbiology, Icahn School of Medicine at Mount Sinai, New York New York.

[2]Global Health and Emerging Pathogens Institute, Icahn School of Medicine at Mount Sinai, New York, NY 10029, USA.

[3]Transplant Immunology Unit, National Center of Microbiology, Instituto de Salud Carlos III, Madrid Spain.

[4]Department of Microbiology, Icahn School of Medicine at Mount Sinai, New York, NY 10029, USA.

[5]Microbiology Section, Dpto. CC, Farmacéuticas y de la Salud, Facultad de Farmacia, Universidad San Pablo-CEU, CEU Universities, 28668, Madrid, Spain.

[6]Institute of Applied Molecular Medicine-Nemesio Díez (IMMA-ND), Department of Basic Medical Sciences, Facultad de Medicina, Universidad San Pablo-CEU, CEU Universities, Urbanización Montepríncipe, 28660 Boadilla del Monte, Madrid, Spain.

[7]Department of Pharmaceutics, Ghent University, Ghent, Belgium.

[8]Graduate School of Biomedical Sciences, Icahn School of Medicine at Mount Sinai, New York, New York, United States of America.

[9]Department of Oncological Sciences, Icahn School of Medicine at Mount Sinai, New York New York.

[10]National Microbiology Center, National Institutes of Health Carlos III, Madrid, Spain.

[11]Icahn Genomics Institute, Icahn School of Medicine at Mount Sinai, New York, New York, United States of America.

[12]Marc and Jennifer Lipschultz Precision Immunology Institute, Icahn School of Medicine at Mount Sinai, New York, New York, United States of America.

SUPPLEMENTARY FIGURES

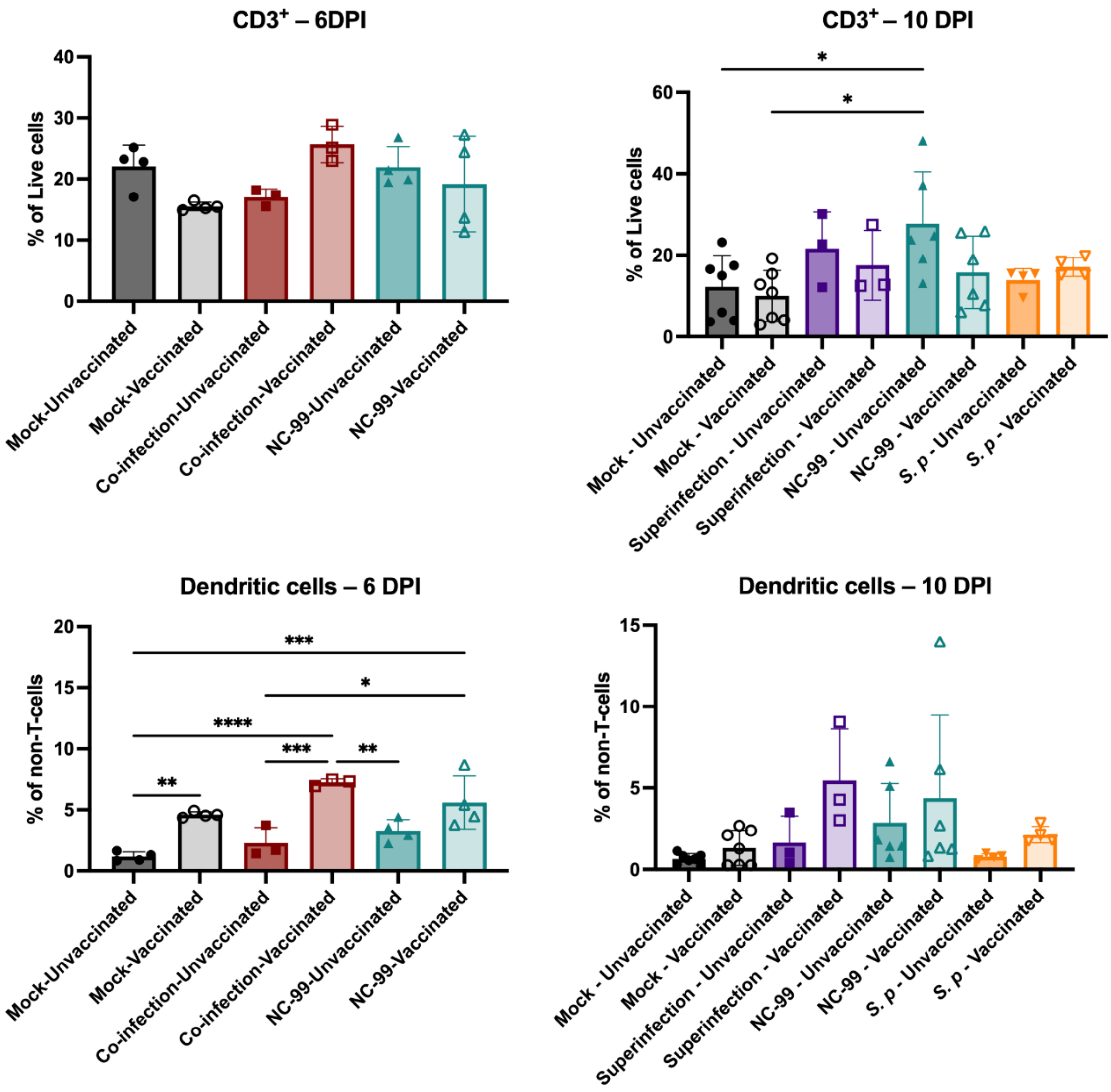

Supplementary Figure 1. Relative quantification of lung CD3+ and dendritic cells at 6 and 10 DPI. Comparisons were performed by One-Way ANOVA with Tukey's multiple comparisons test. Significance values are represented as * p≤0.05, ** p≤0.01, *** p≤0.001, **** p≤0.0001.

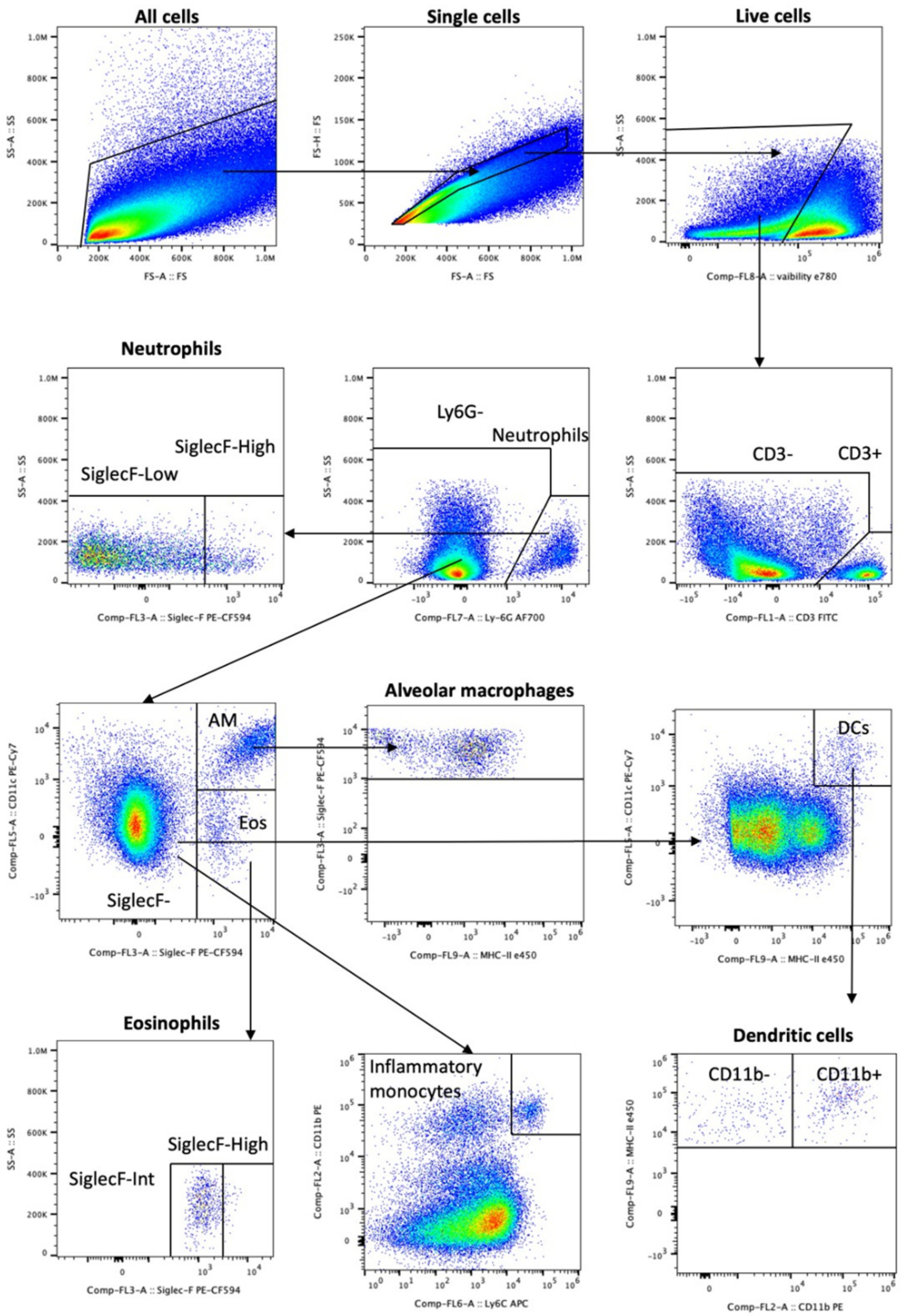

Supplementary Figure 2. Gating strategy for classification of lung cell populations. Gating strategy, exemplified in an influenza NC99 infected lung at 10 DPI.

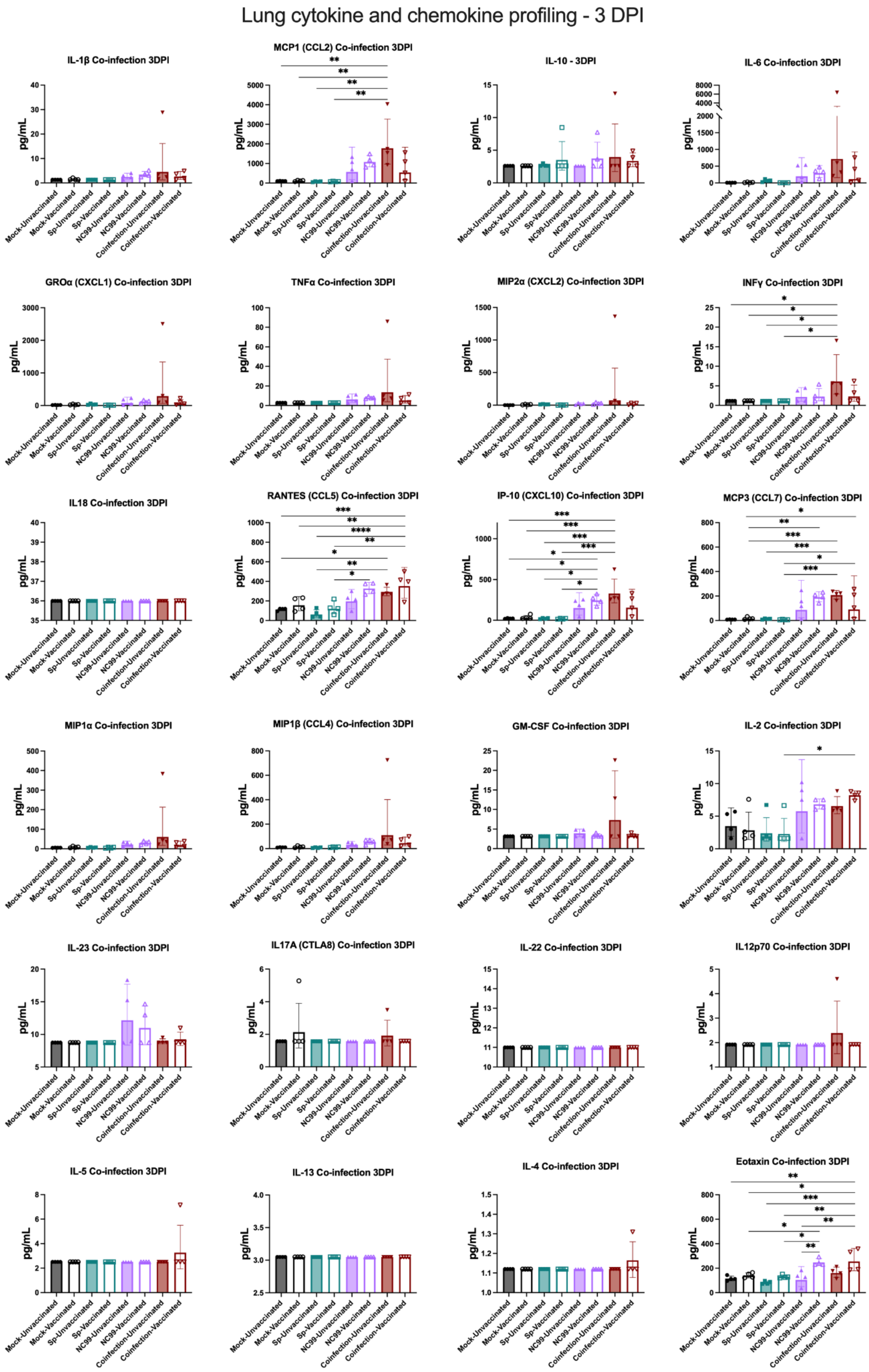

Supplementary Figure 3. lung homogenate supernatant cytokine/chemokine data at 3 DPI. Bar graphs (Mean±SD) of absolute concentration (pg/ml) of cytokines and chemokines in lungs.

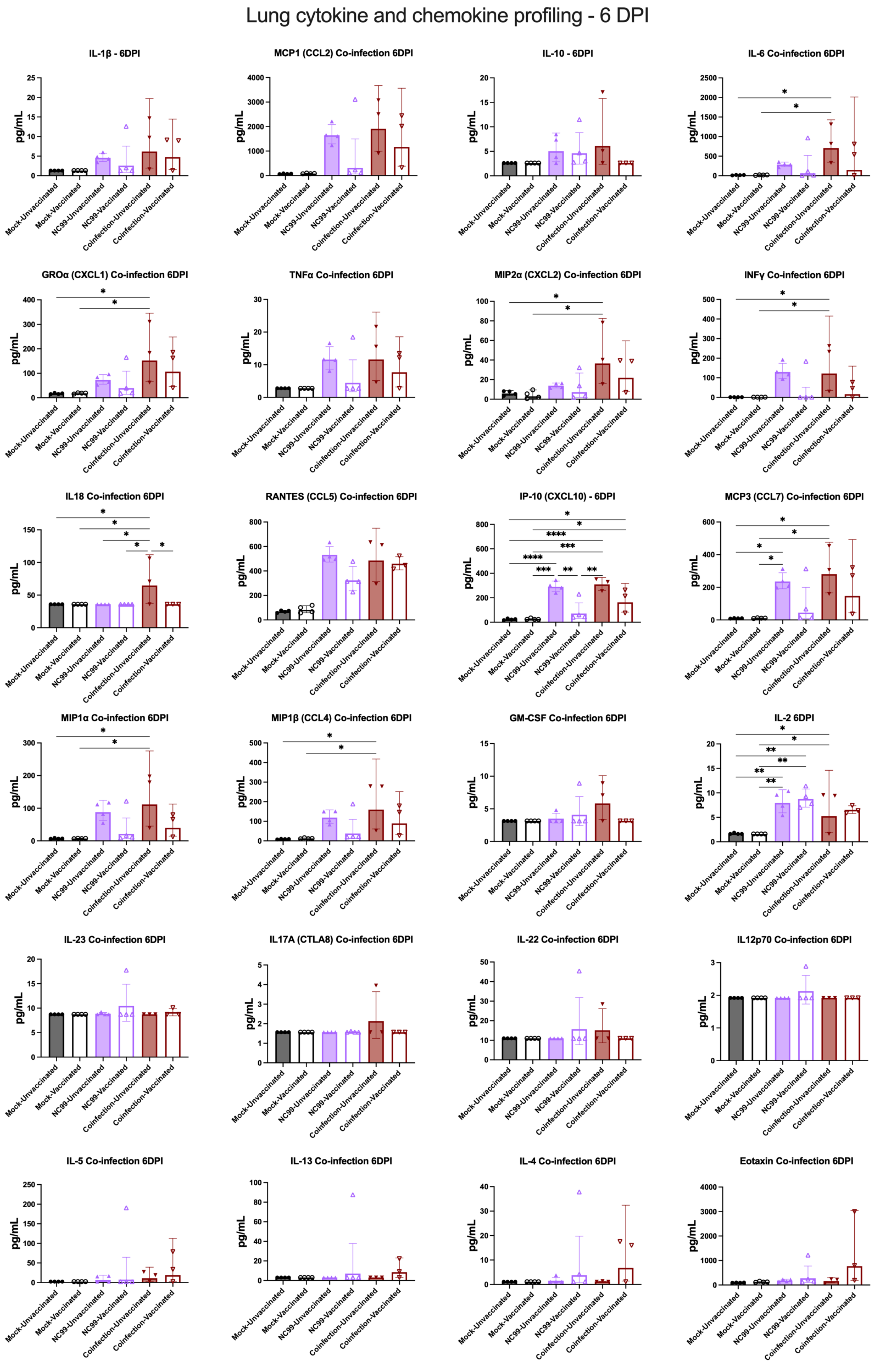

Supplementary Figure 4. lung homogenate supernatant cytokine/chemokine data at 6 DPI. Bar graphs (Mean±SD) of absolute concentration (pg/ml) of cytokines and chemokines in lungs.

Lung cytokine and chemokine profiling - 10 DPI

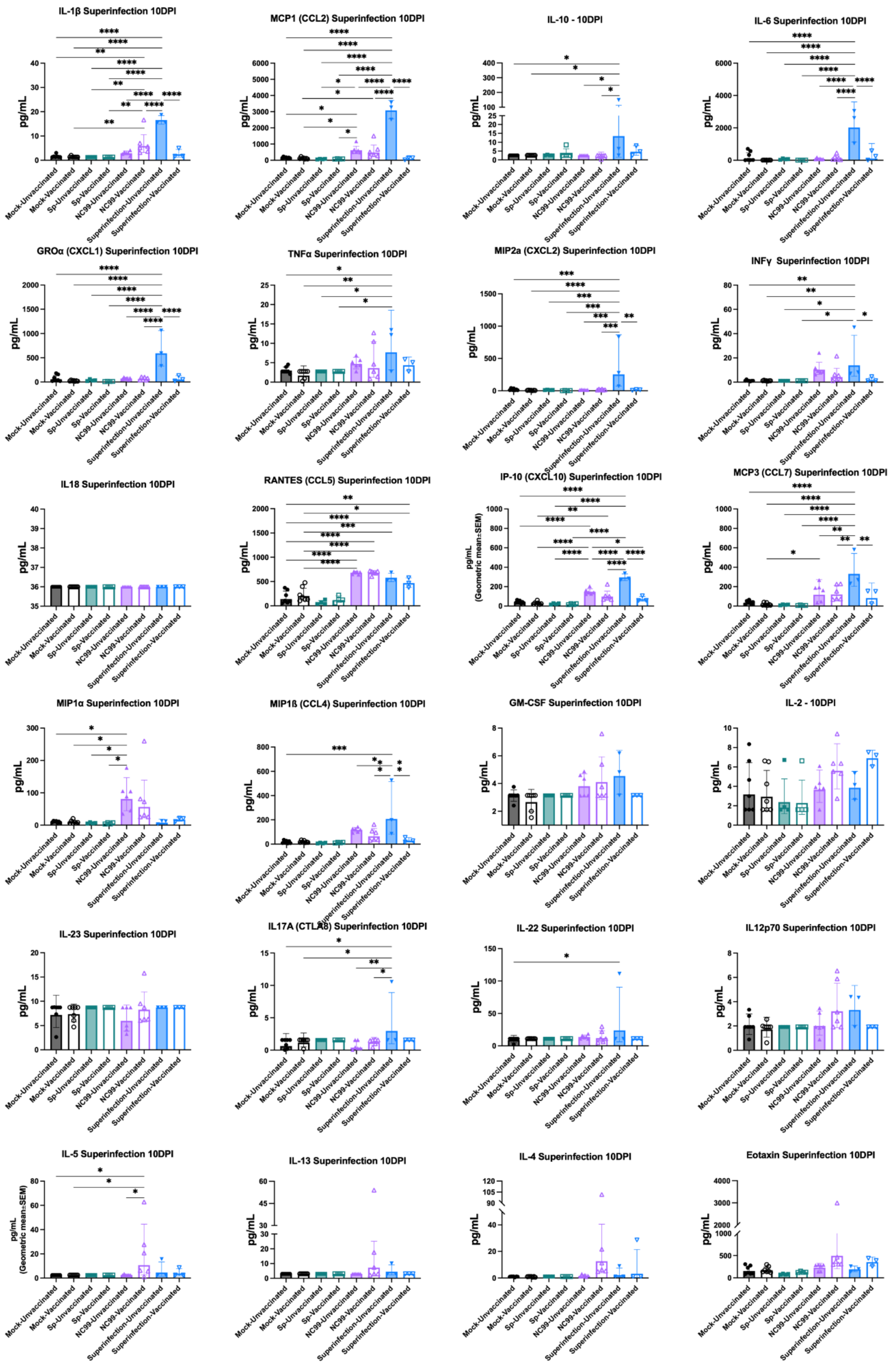

Supplementary Figure 5. lung homogenate supernatant cytokine/chemokine data at 10 DPI. Bar graphs (Mean±SD) of absolute concentration (pg/ml) of cytokines and chemokines in lungs.
